# Supplementary material for: CRL2ZER1/ZYG11B recognizes small N-terminal residues for degradation
Source: Nat Commun. 2022 Dec 10;13:7636. doi: 10.1038/s41467-022-35169-6 (PMC9741652; doi:10.1038/s41467-022-35169-6)
Supplement: Supplementary file 1 — supplementary information file [file 41467_2022_35169_MOESM1_ESM.pdf]

Supplementary information for

CRL2$^{ZER1/ZYG11B}$ recognizes small N-terminal residues for degradation

Yao Li[1#], Yueling Zhao[2#], Xiaojie Yan[1#], Chen Ye[2#], Sara Weirich[3], Bing Zhang[1], Xiaolu Wang[4], Lili Song[2], Chenhao Jiang[2], Albert Jeltsch[3], Cheng Dong[1*] and Wenyi Mi[2*]

*Corresponding author. Email: dongcheng@tmu.edu.cn or wenyi.mi@tmu.edu.cn

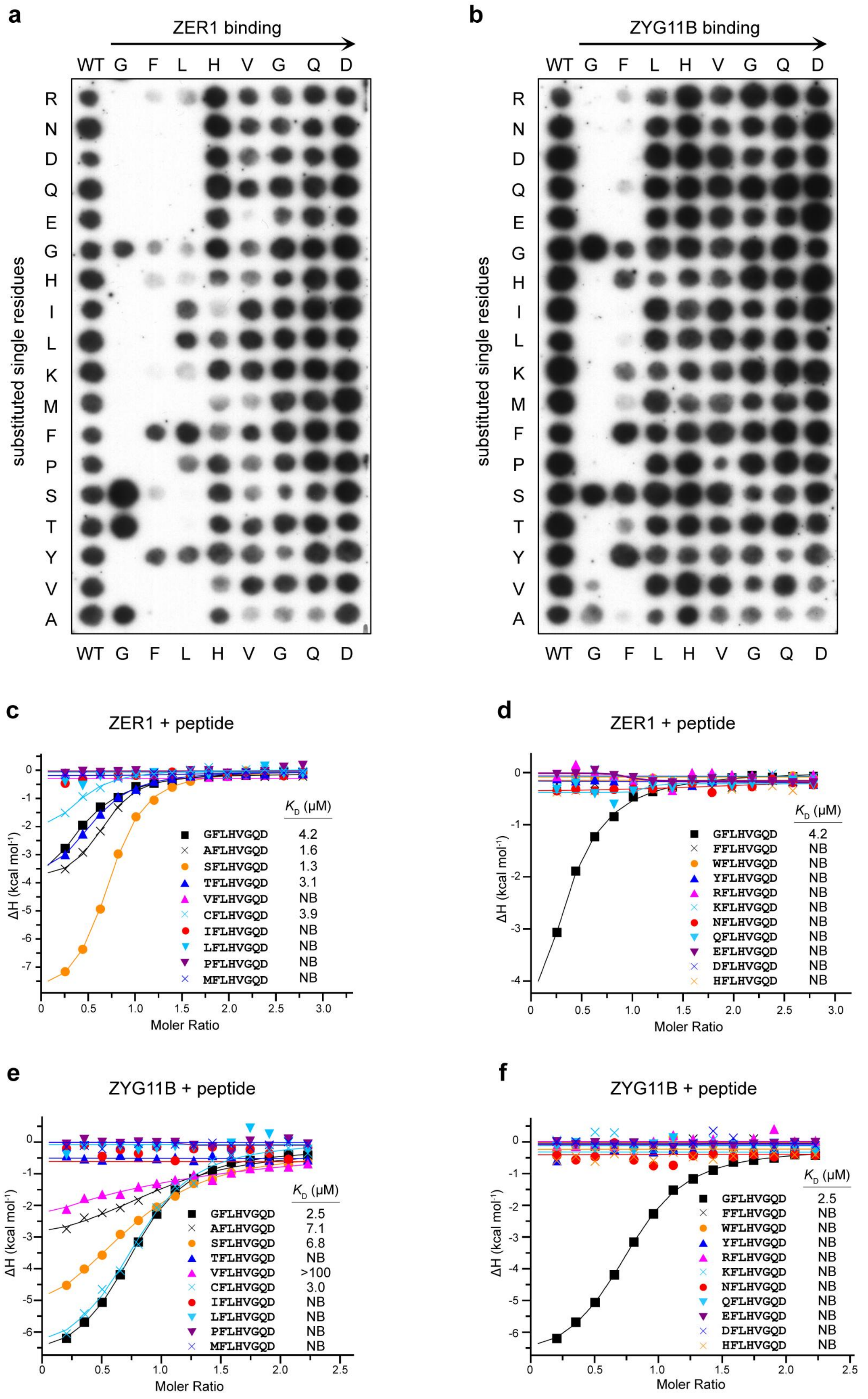

Supplementary Fig. 1 | Interactions of XFLHVGQD (X is the set of 20 amino acids in the genetic code) peptide with human ZER1 and ZYG11B. a-b Binding patterns of GST-tagged $ZER1_{469–766}$ and $ZYG11B_{490–744}$ to an array of the spotted GFLHVGQDGLELPTS peptide (top row,

denoted “wild-type” [WT]) and its indicated derivatives containing mutations in the first 8 positions. Representative images, n=3. **c-d** ITC fitting curves of ZER1 (residues 469–766) titrated by XFLHVGQD peptides. **e-f** ITC fitting curves of ZYG11B (residues 490–728) titrated by a series of XFLHVGQD peptides. The corresponding peptide sequences and binding affinities are indicated. NB, no detectable binding.

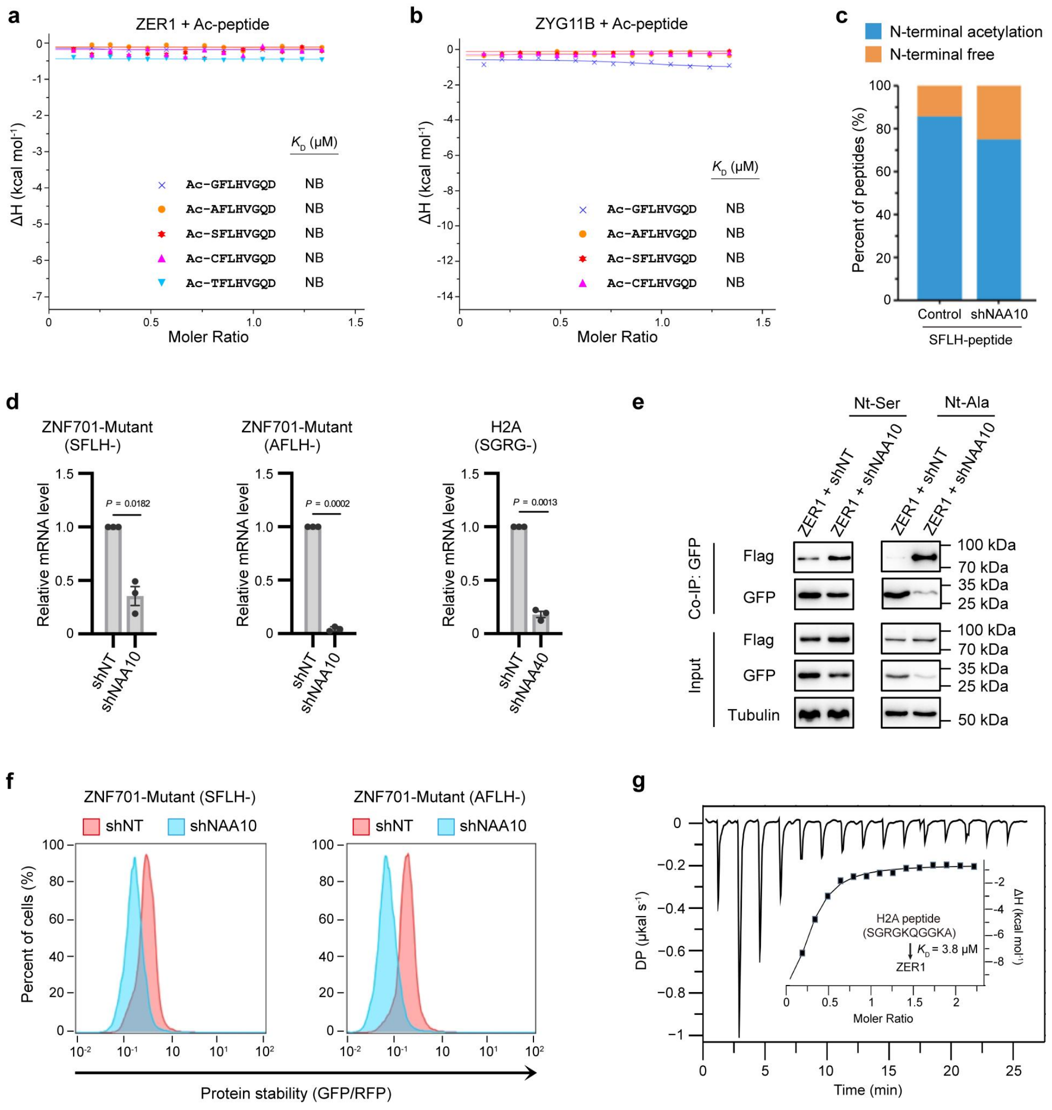

Supplementary Fig. 2 | Nt-Ac shields the substrates from recognition by ZER1. a-b ITC measurements of the interactions of ZER1 (residues 469–766) or ZYG11B (residues 490–728) with Nt-Ac peptides. NB, no detectable binding. **c** LC-MS analysis of acetylation levels of Ub-SFLH-GFP in the NAA10-knockdown cells. **d** The relative mRNA levels of NAA10 or NAA40 in their knockdown cells. Error bars represent the standard error of mean (s.e.m.). Data are mean ± s.e.m.. *P* values were determined using unpaired two-tailed Student's t-tests; n=3 biologically independent samples. **e** Co-IP analysis of the interactions of full-length ZER1 with SFLH- or AFLH-fused GFP. HEK293T cells expressing Ub-SFLH-GFP or Ub-AFLH-GFP were infected with Flag-tagged full-length ZER1 lentivirus, then the infected cells were selected for stable cells, which were subsequently infected by NAA10 shRNA lentivirus. Cells were collected and used for Co-IP experiments. Representative images, n=3. Uncropped western blot images are provided in Source data 1. **f** Stability analysis of SFLH-GFP or AFLH-GFP in NAA10 knockdown cell lines. The ratio of GFP/RFP was analyzed by flow cytometry. FACS sequential gating strategies are provided in Supplementary Fig. 6. **g** ITC titration and fitting curves of ZER1 (residues 469–766) binding to the H2A histone peptide.

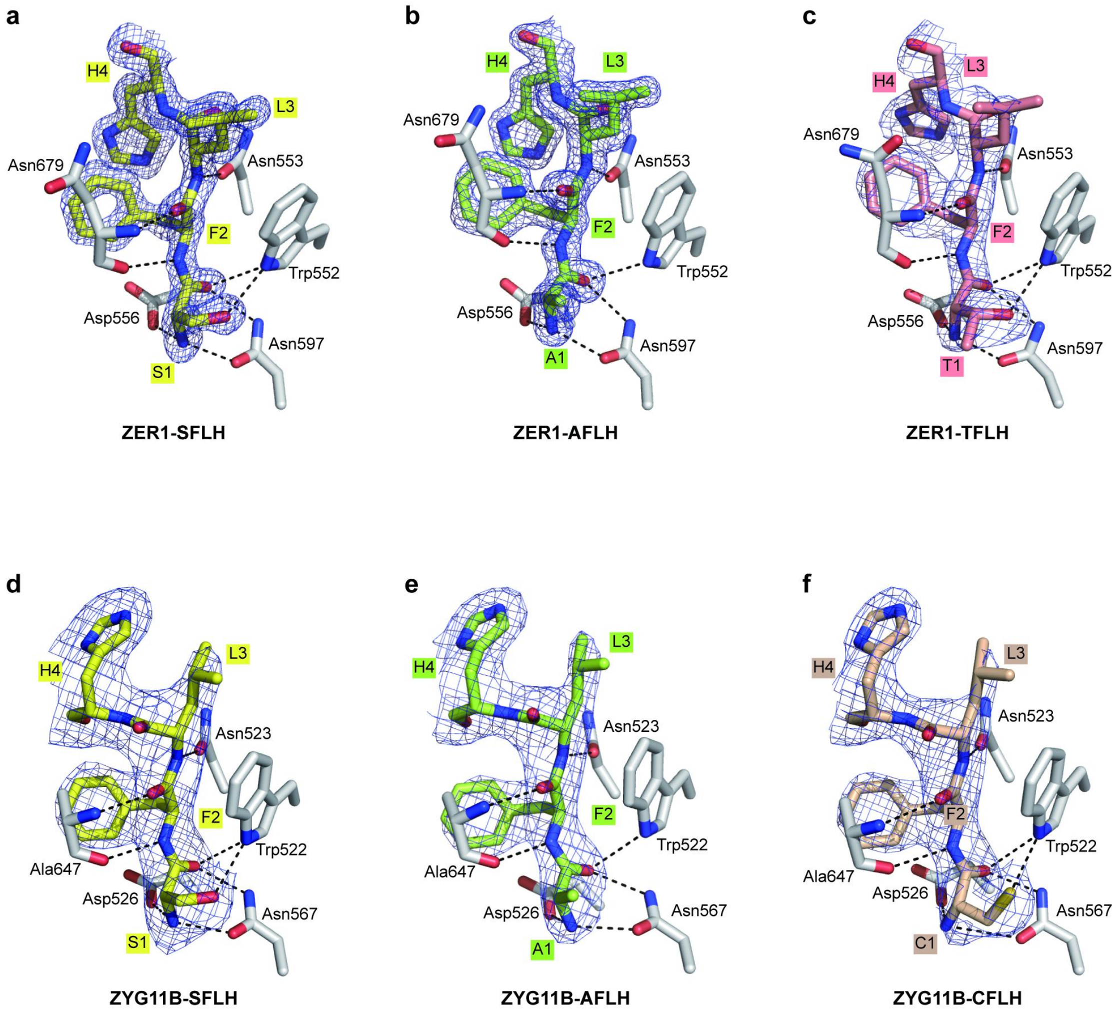

Supplementary Fig. 3 | Structural details of different peptides bound to ZER1 and ZYG11B. The peptides are depicted as yellow (SFLH), palegreen (AFLH), pink (TFLH) and palebrown (CFLH) sticks. ZER1 and ZYG11B are depicted as gray sticks. **a** SFLH in ZER1-SFLH complex; **b** AFLH in ZER1-AFLH complex; **c** TFLH in ZER1-TFLH complex; **d** SFLH in ZYG11B-SFLH complex; **e** AFLH in ZYG11B-AFLH complex; **f** CFLH in ZYG11B-CFLH complex. 2Fo–Fc electron density maps (blue mesh) of the peptides are contoured at the 1.0 - 1.5σ level.

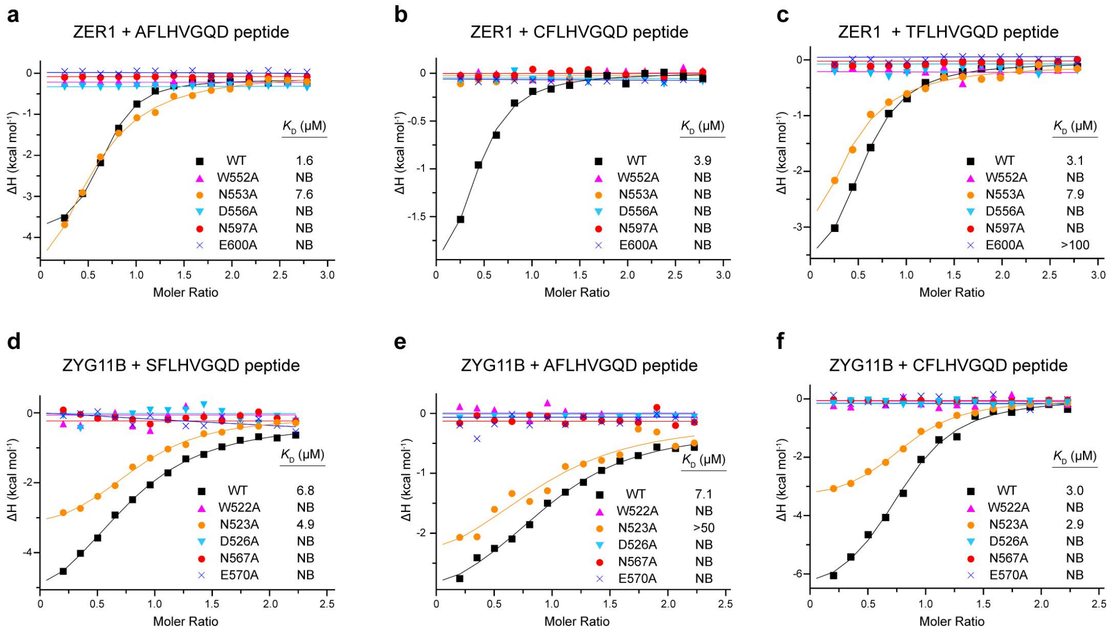

Supplementary Fig. 4 | ITC measurements of wild-type (WT) and mutant proteins binding to XFLHVGQD. a-c Binding affinities of ZER1 with XFLHVGQD (X = A, C or T); **d-f** Binding affinities of ZYG11B with XFLHVGQD (X = S, A or C). The corresponding mutations and binding affinities (K_Ds) are indicated. NB, no detectable binding.

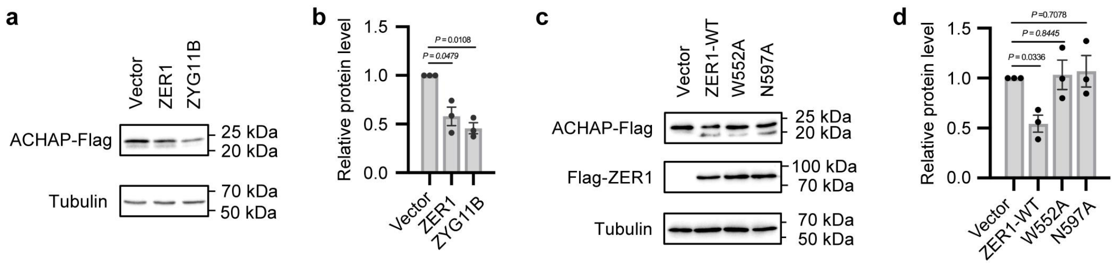

Supplementary Fig. 5 | Western blotting analysis of ACHAP (acetylcholinesterase-associated protein) stability. **a** Stability analysis of ACHAP full-length protein with overexpression of ZER1 or ZYG11B in HEK293T cells by western blotting. Representative images, n=3. **b** The relative protein levels of ACHAP were quantified with Gel Image System (Tanon-5200) and normalized to α-tubulin according to the results of three experiments. Error bars represent the standard error of mean (s.e.m.). Data are mean ± s.e.m.. *P* values were determined using unpaired two-tailed Student's t-tests; n=3 biologically independent samples. **c** Stability analysis of ACHAP full-length protein with overexpression of WT and indicated mutant ZER1 proteins in HEK293T cells by western blotting. Representative images, n=3. **d** The relative protein levels of ACHAP were quantified with Gel Image System (Tanon-5200) and normalized to α-tubulin according to the results of three experiments. Error bars represent the standard error of mean (s.e.m.). Data are mean ± s.e.m.. *P* values were determined using unpaired two-tailed Student's t-tests; n=3 biologically independent samples. Uncropped western blot images are provided in Source data 1.

For Fig. 2b
ZNF701-WT (GFLH-)

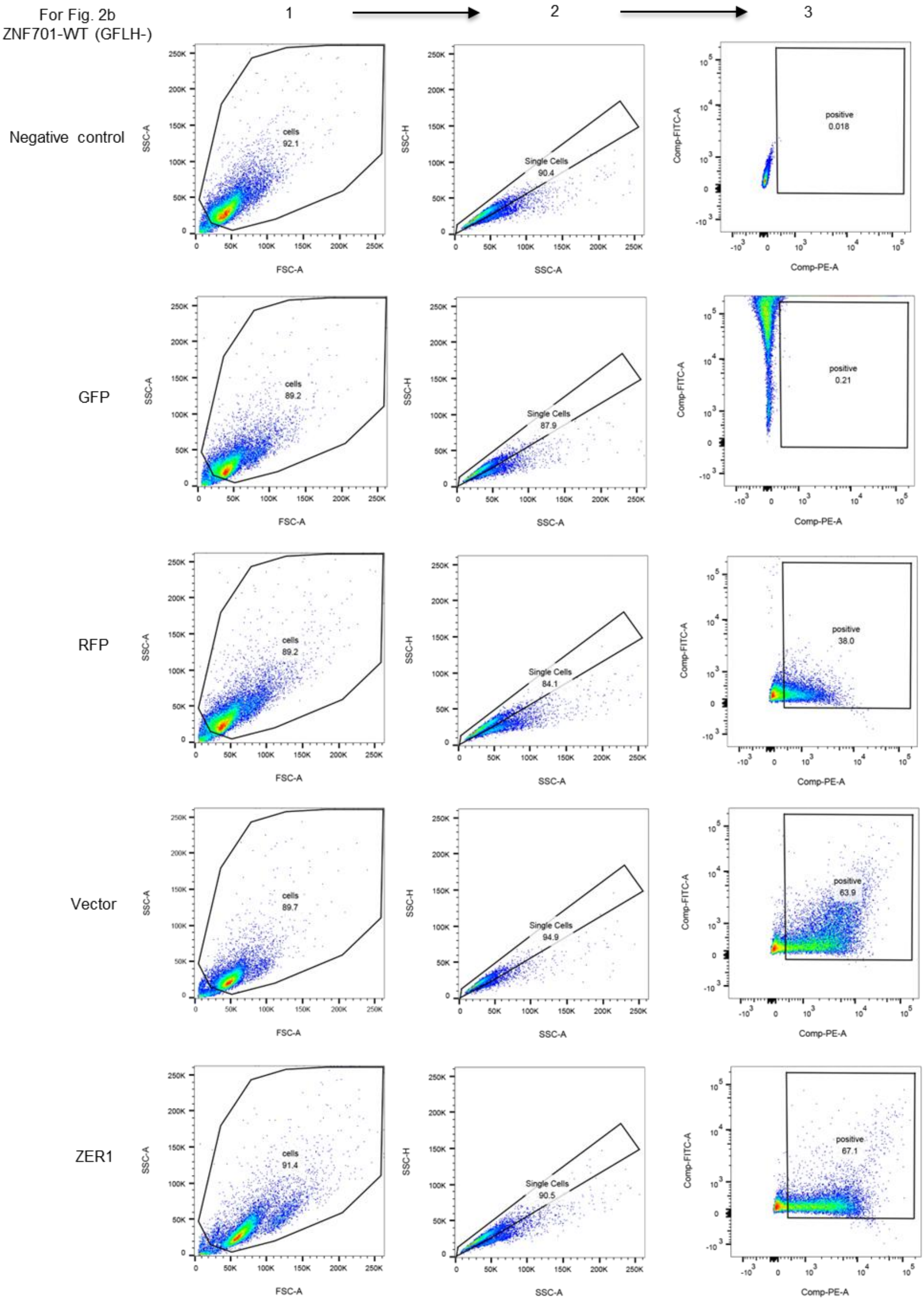

For Fig. 2b
ZNF701-Mutant (SFLH-)

Negative control

GFP

RFP

Vector

ZER1

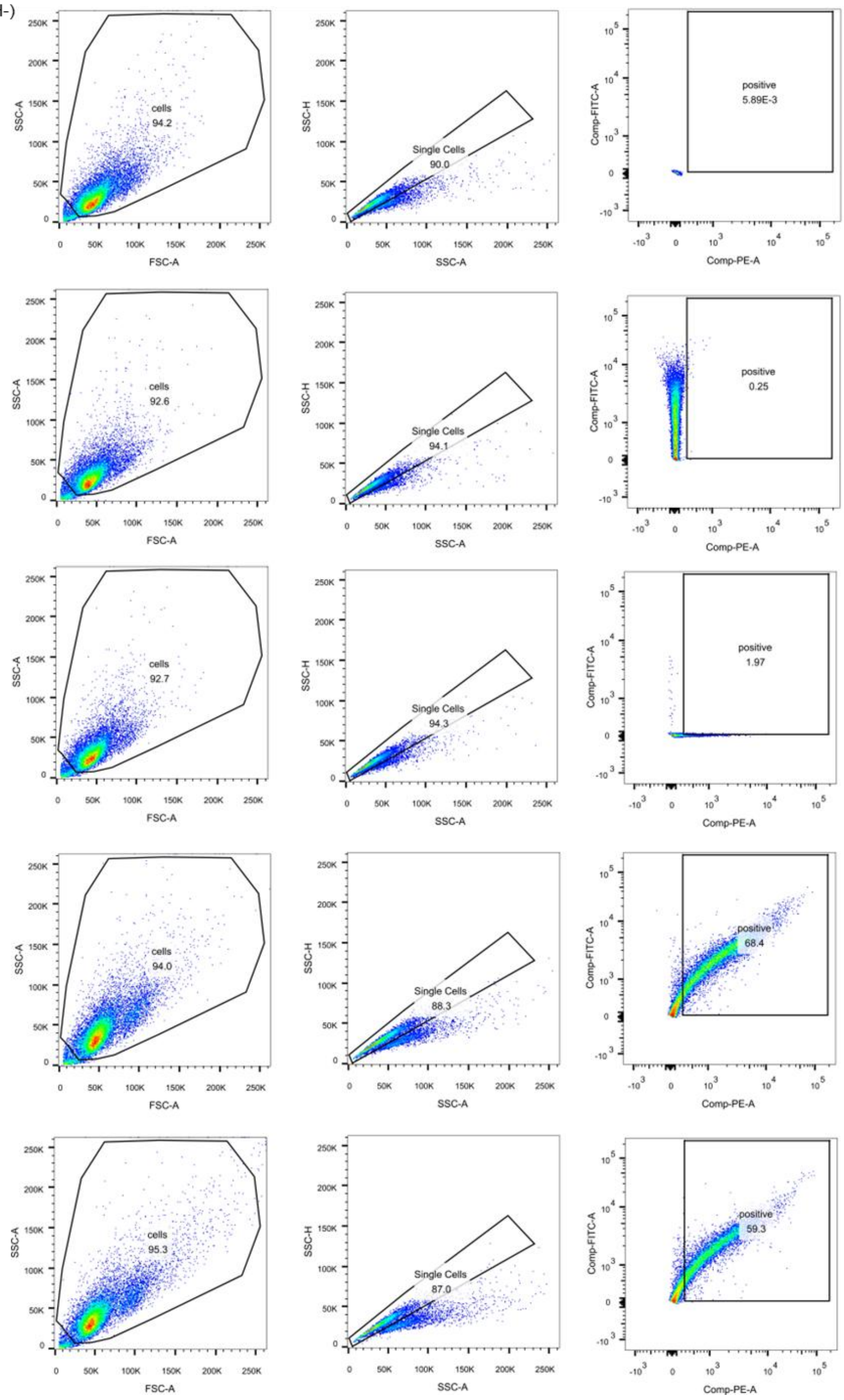

For Fig. 2b
ZNF701-Mutant (AFLH-)

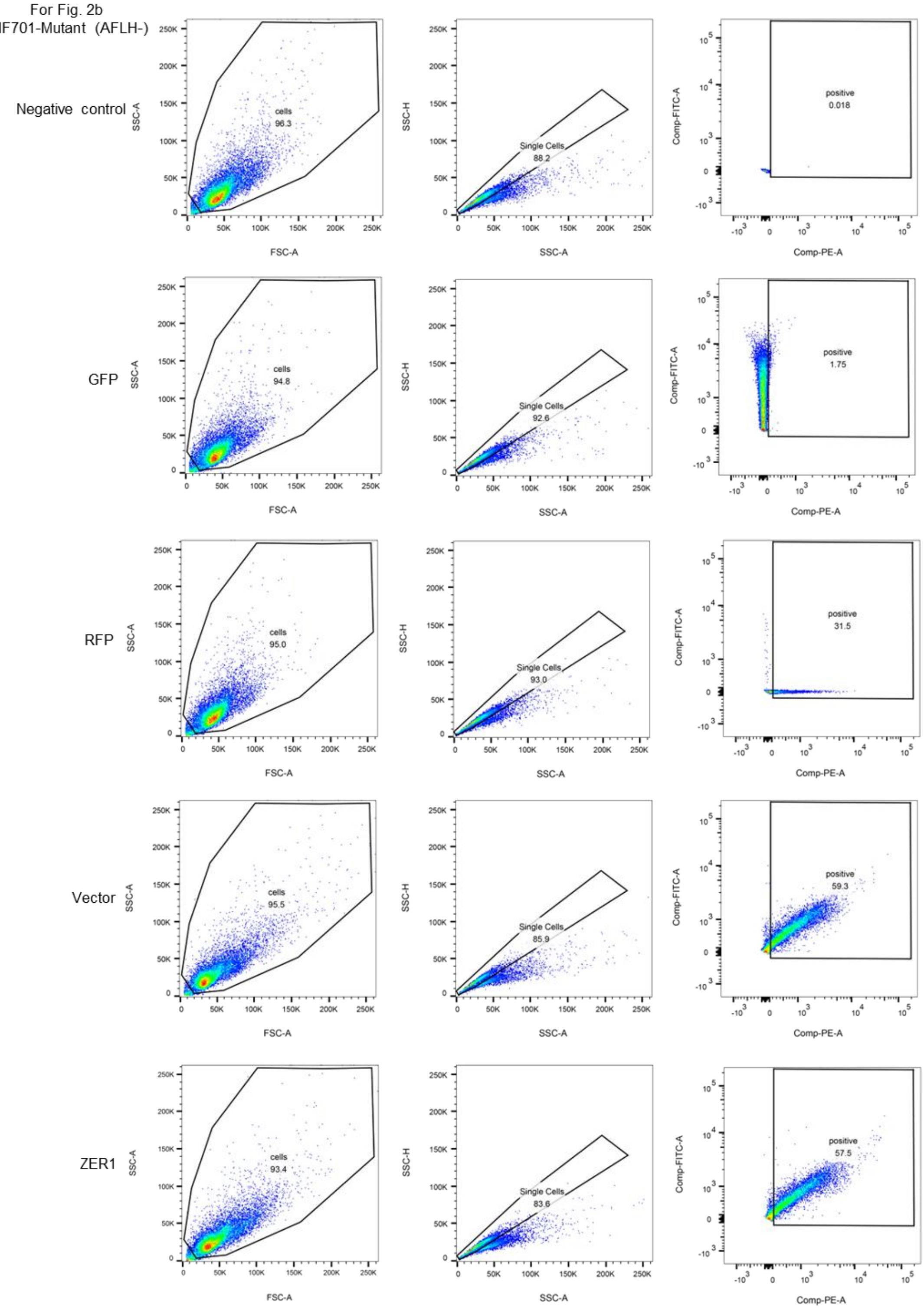

For Fig. 2c
ZNF701-Mutant (SFLH-)

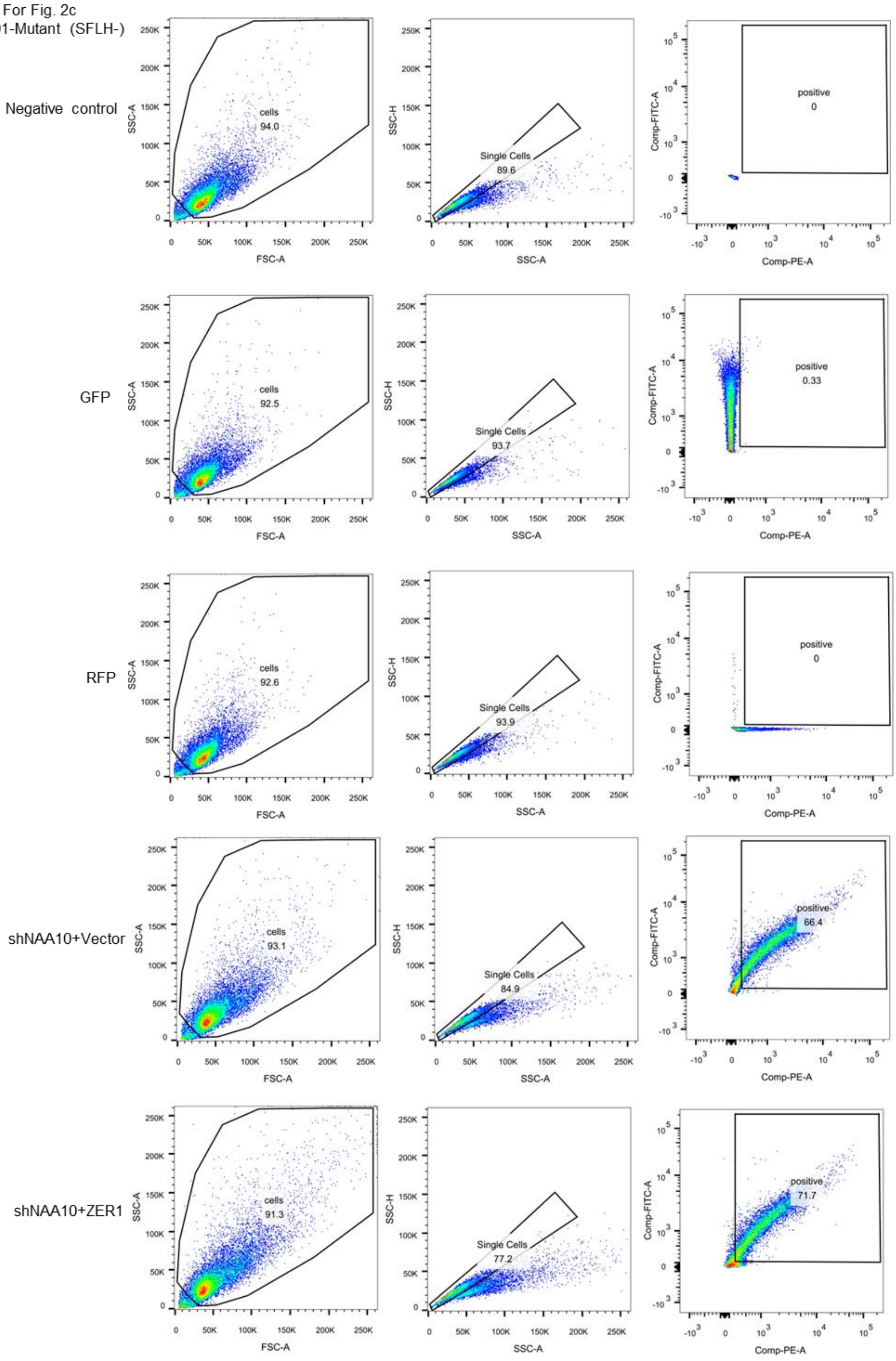

For Fig. 2c
ZNF701-Mutant (AFLH-)

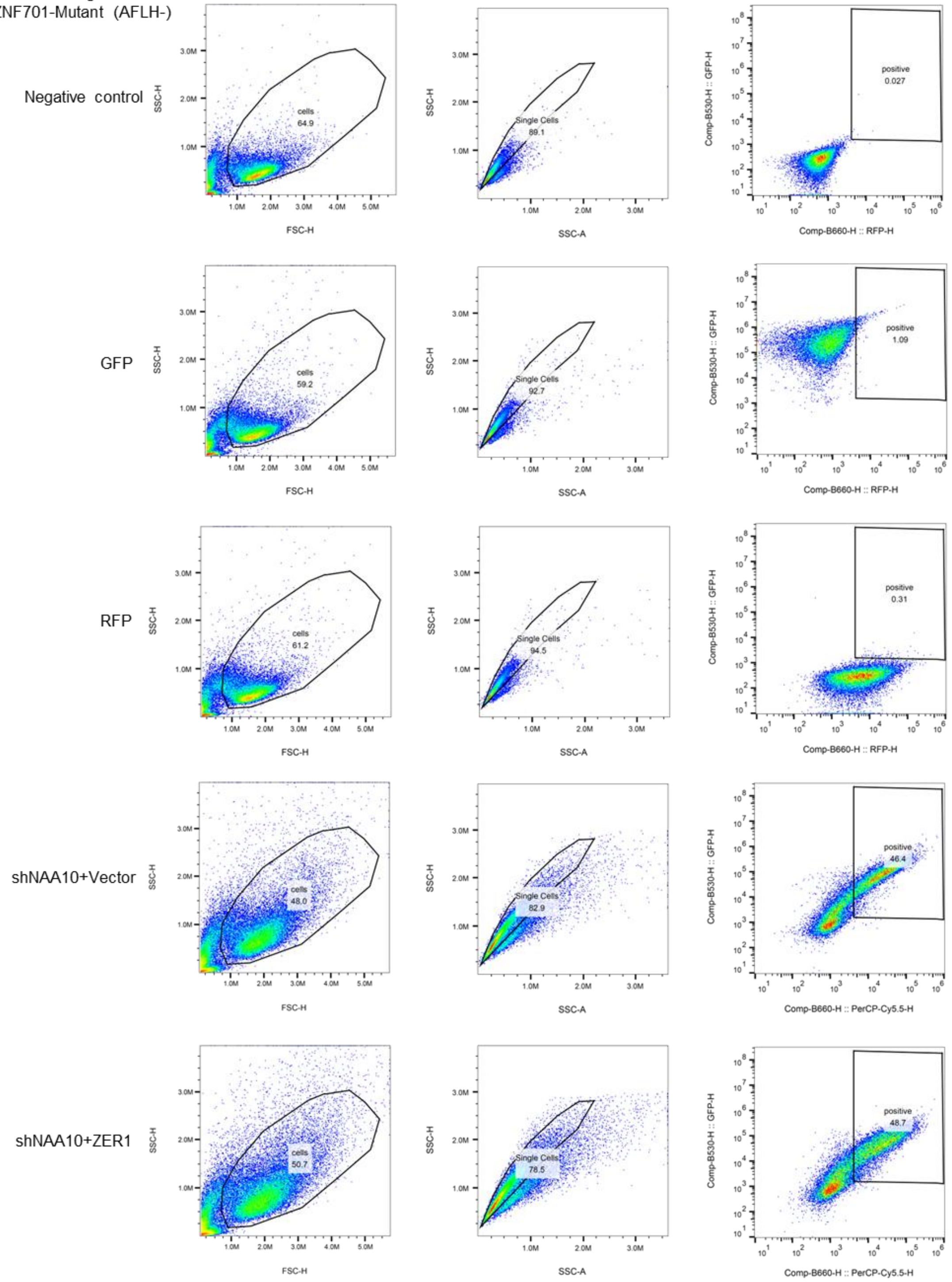

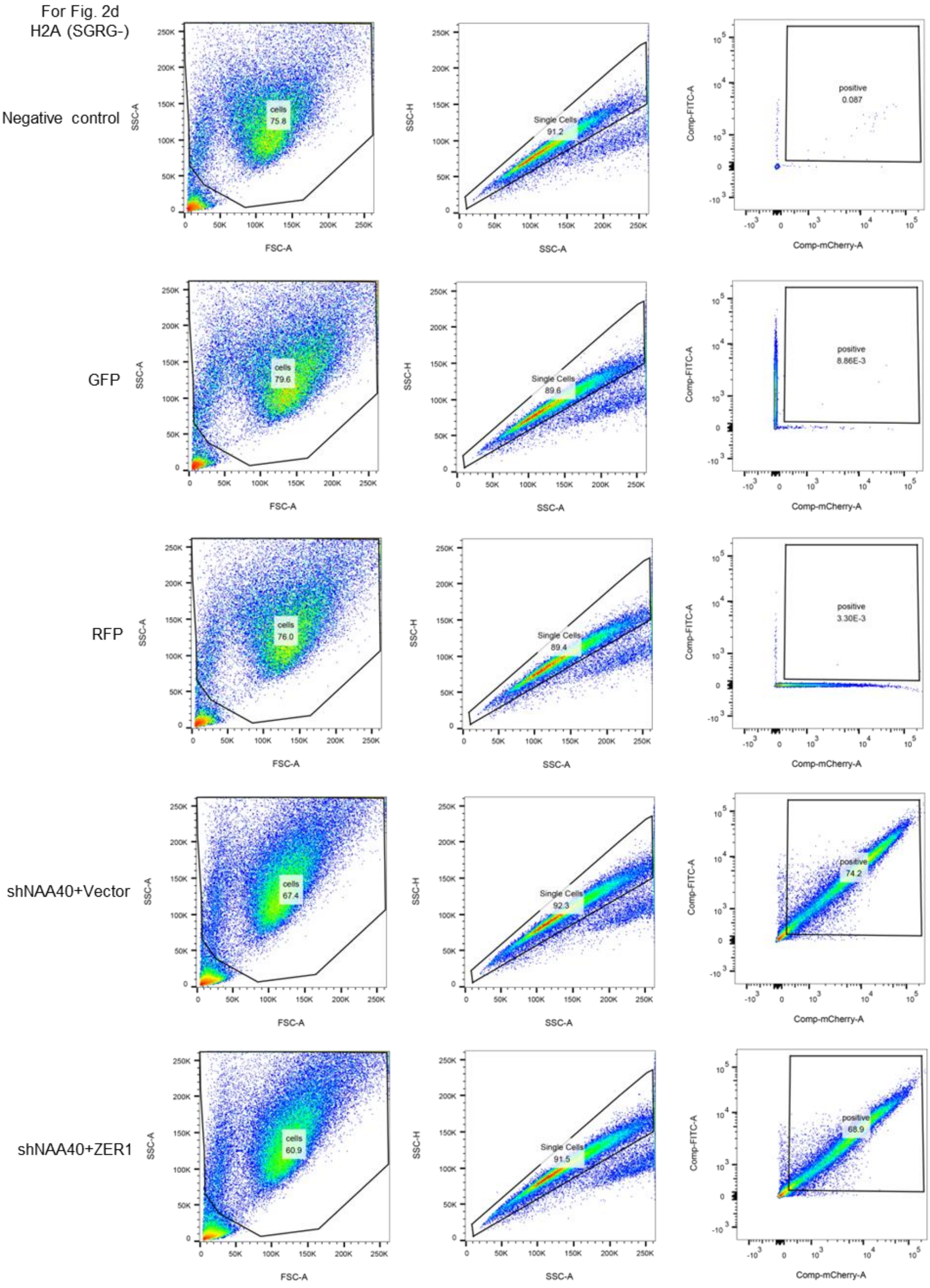
For Fig. 2d
H2A (SGRG-)
Negative control
GFP
RFP
shNAA40+Vector
shNAA40+ZER1
SSC-A
FSC-A
SSC-H
Comp-FITC-A
Comp-mCherry-A
cells
75.8
79.6
76.0
67.4
60.9
Single Cells
91.2
89.6
89.4
92.3
91.5
positive
0.087
8.86E-3
3.30E-3
74.2
68.9

For Fig. 2e
ZNF701-Mutant (CFLH-)

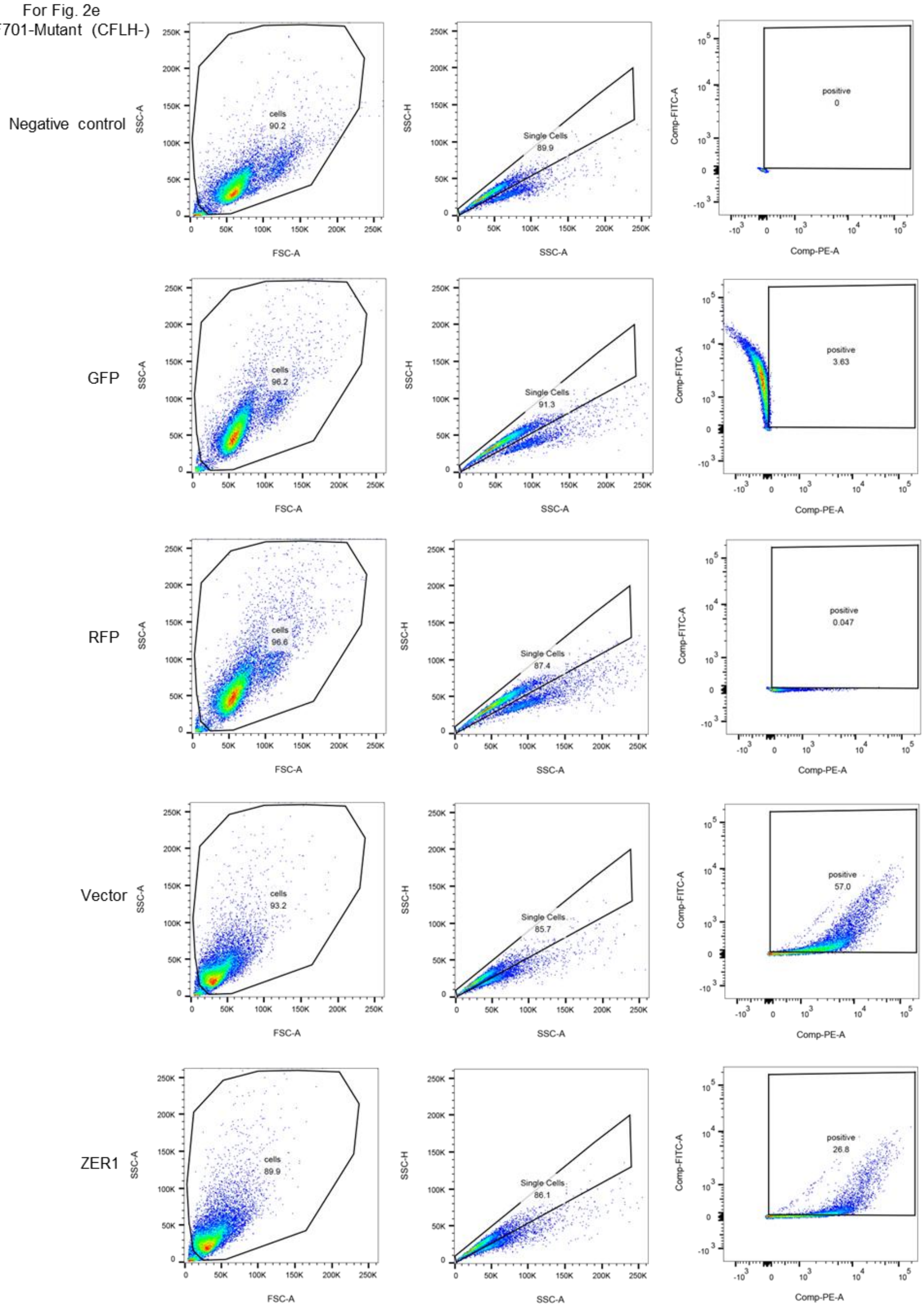

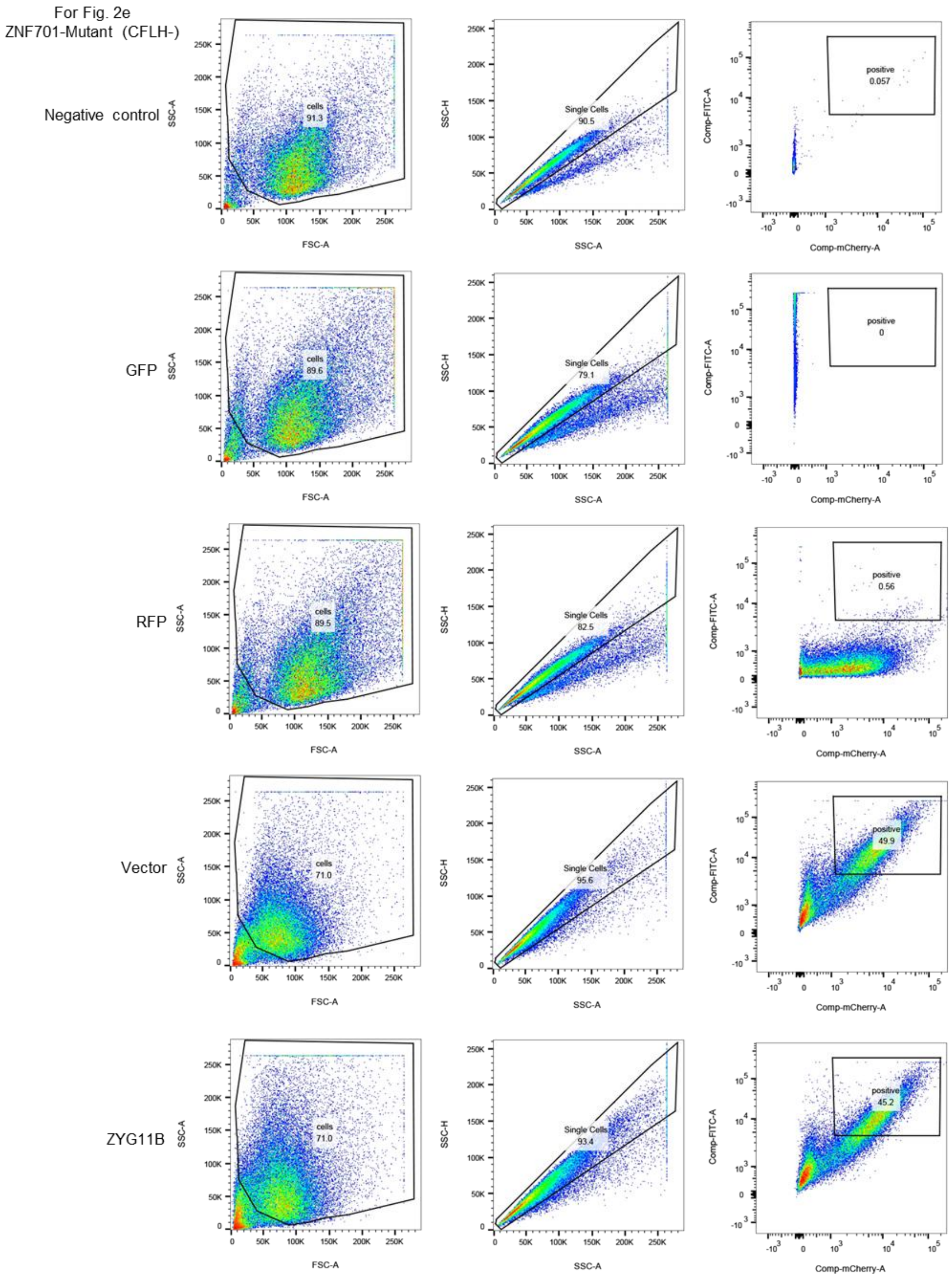
For Fig. 2e
ZNF701-Mutant (CFLH-)
Negative control
GFP
RFP
Vector
ZYG11B
cells 91.3
cells 89.6
cells 89.5
cells 71.0
cells 71.0
Single Cells 90.5
Single Cells 79.1
Single Cells 82.5
Single Cells 95.6
Single Cells 93.4
positive 0.057
positive 0
positive 0.56
positive 49.9
positive 45.2
FSC-A
SSC-A
SSC-H
Comp-FITC-A
Comp-mCherry-A

For Fig. 2f
ZNF701-Mutant (CFLH-)

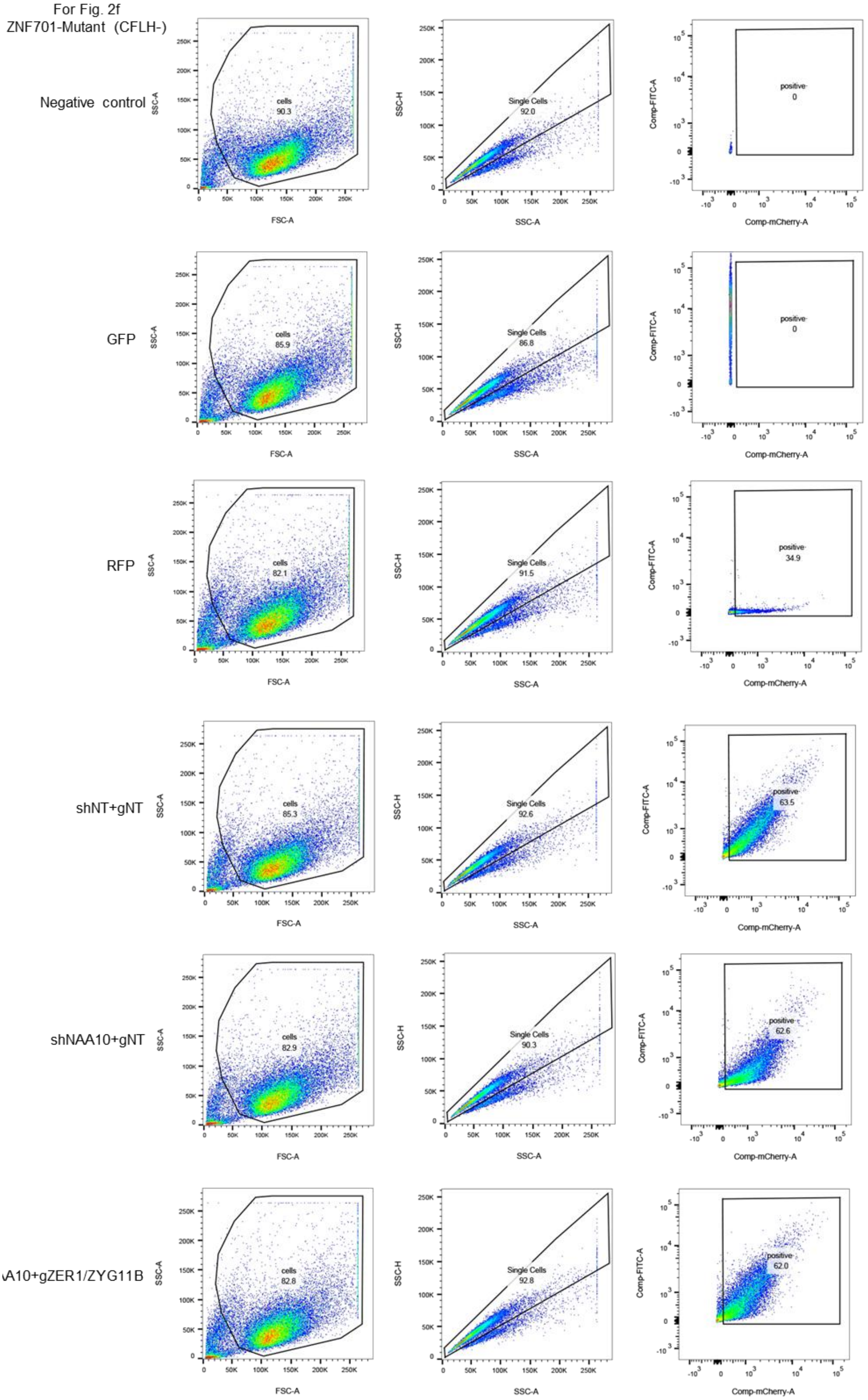

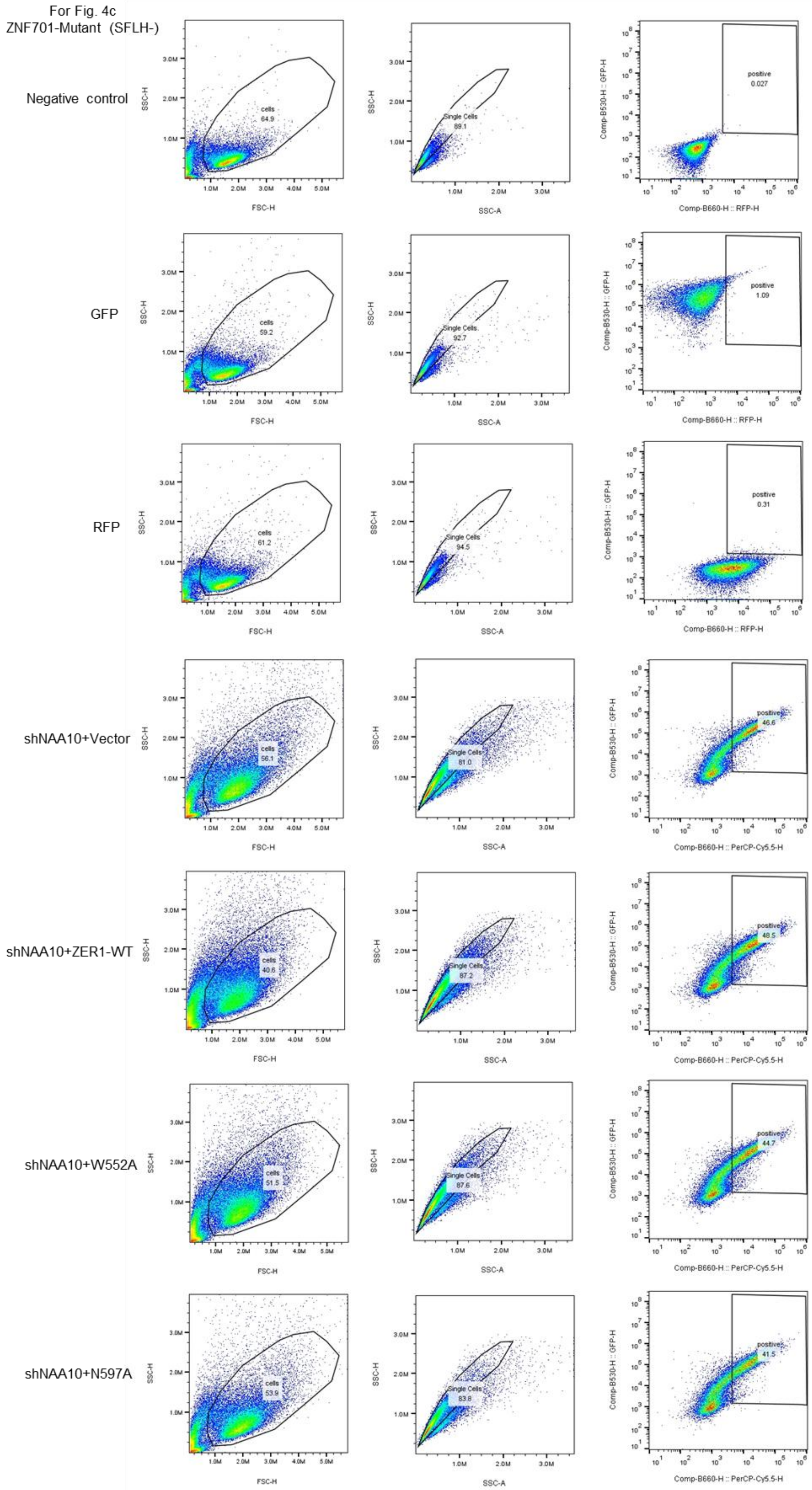

For Fig. 4c
ZNF701-Mutant (SFLH-)
Negative control
cells 64.9
Single Cells 89.1
positive 0.027
GFP
cells 59.2
Single Cells 92.7
positive 1.09
RFP
cells 61.2
Single Cells 94.5
positive 0.31
shNAA10+Vector
cells 56.1
Single Cells 81.0
positive 46.6
shNAA10+ZER1-WT
cells 40.6
Single Cells 87.2
positive 48.5
shNAA10+W552A
cells 51.5
Single Cells 87.6
positive 44.7
shNAA10+N597A
cells 53.9
Single Cells 83.8
positive 41.5
SSC-H
FSC-H
SSC-A
Comp-B530-H :: GFP-H
Comp-B660-H :: RFP-H
Comp-B660-H :: PerCP-Cy5.5-H

For Fig. 4e
ZNF701-Mutant (AFLH-)

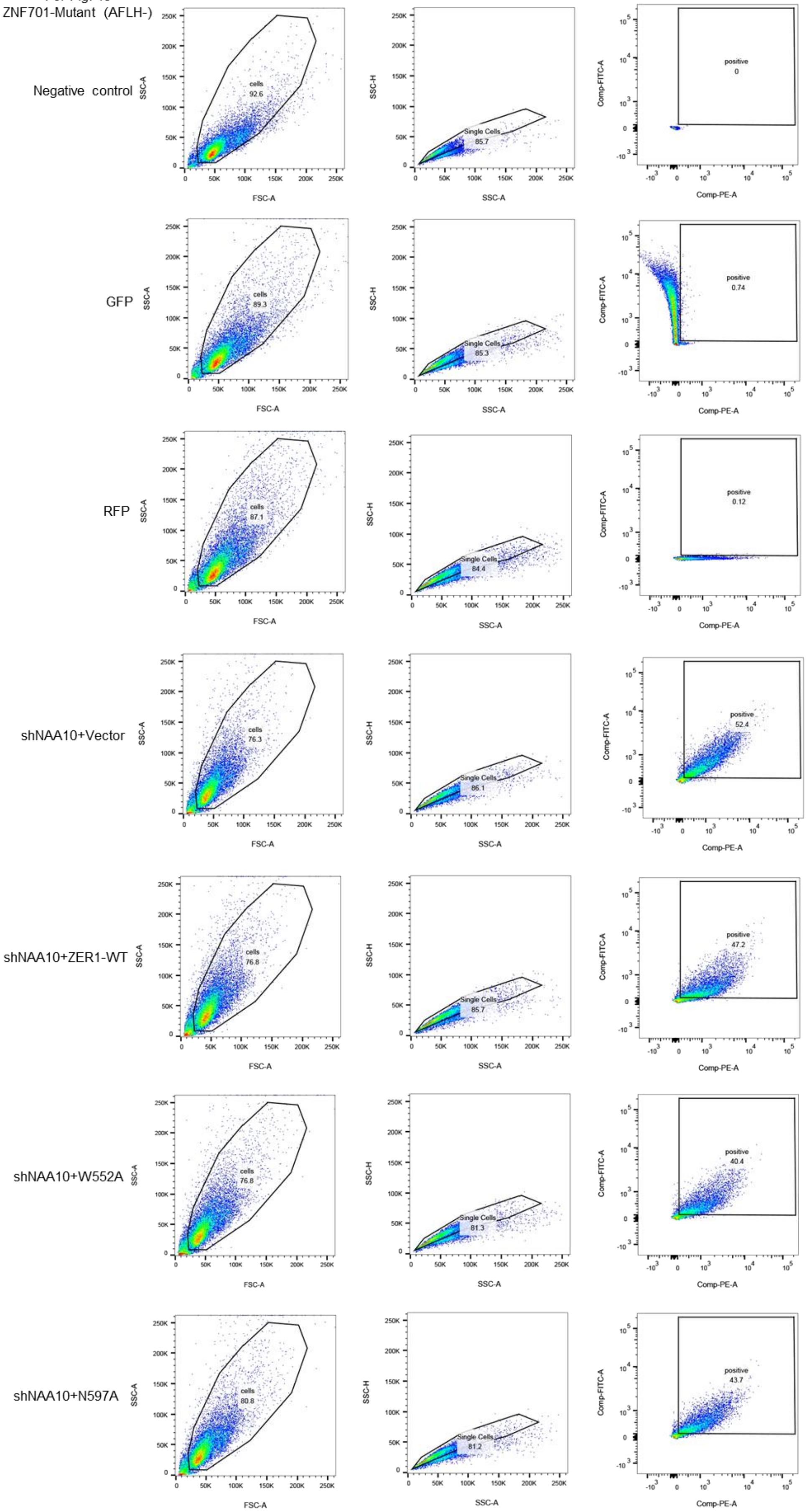

For Fig. 4e
H2A (SGRG-)

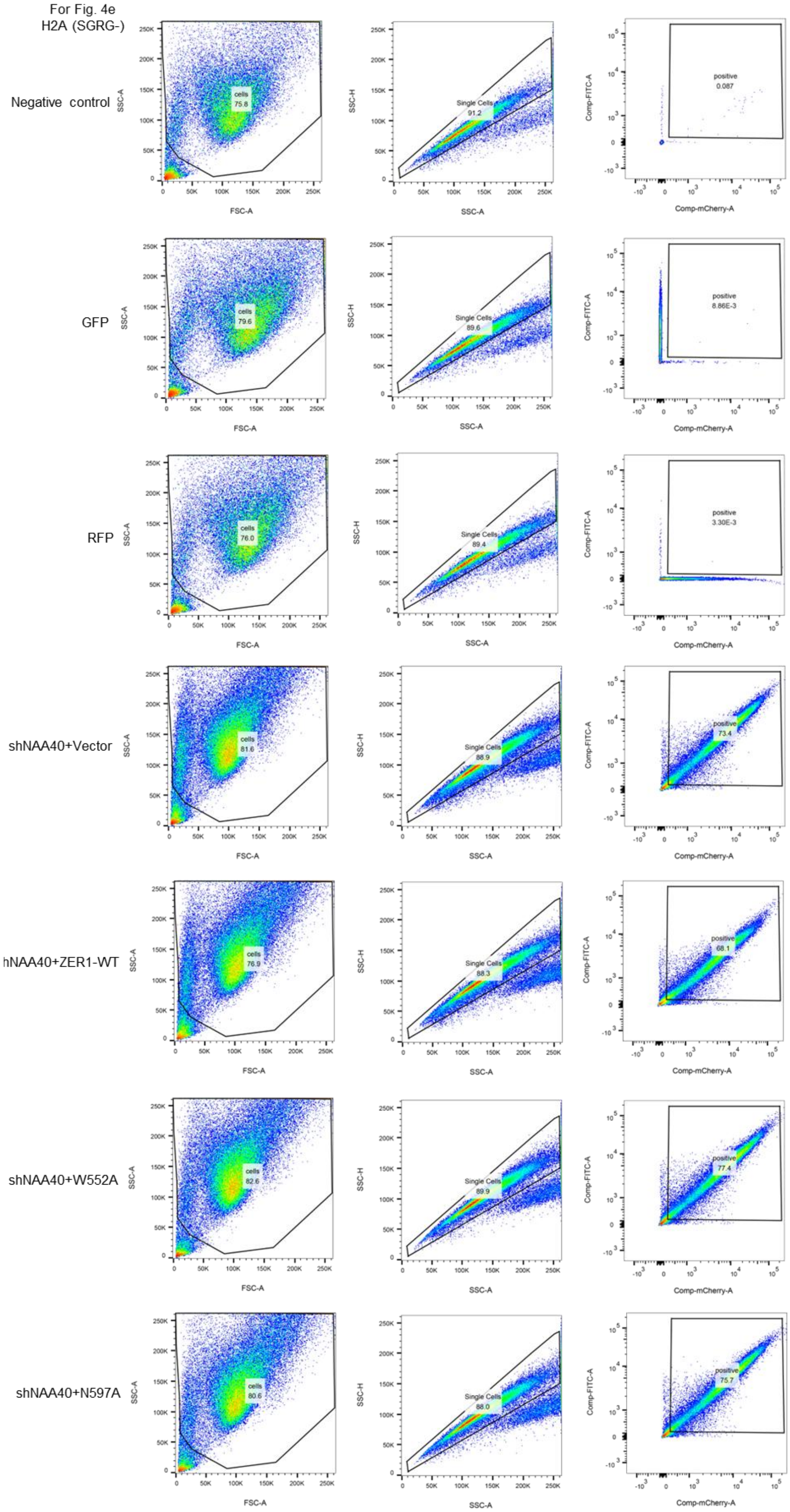

For Fig. 4i
ZNF701-Mutant (CFLH-)

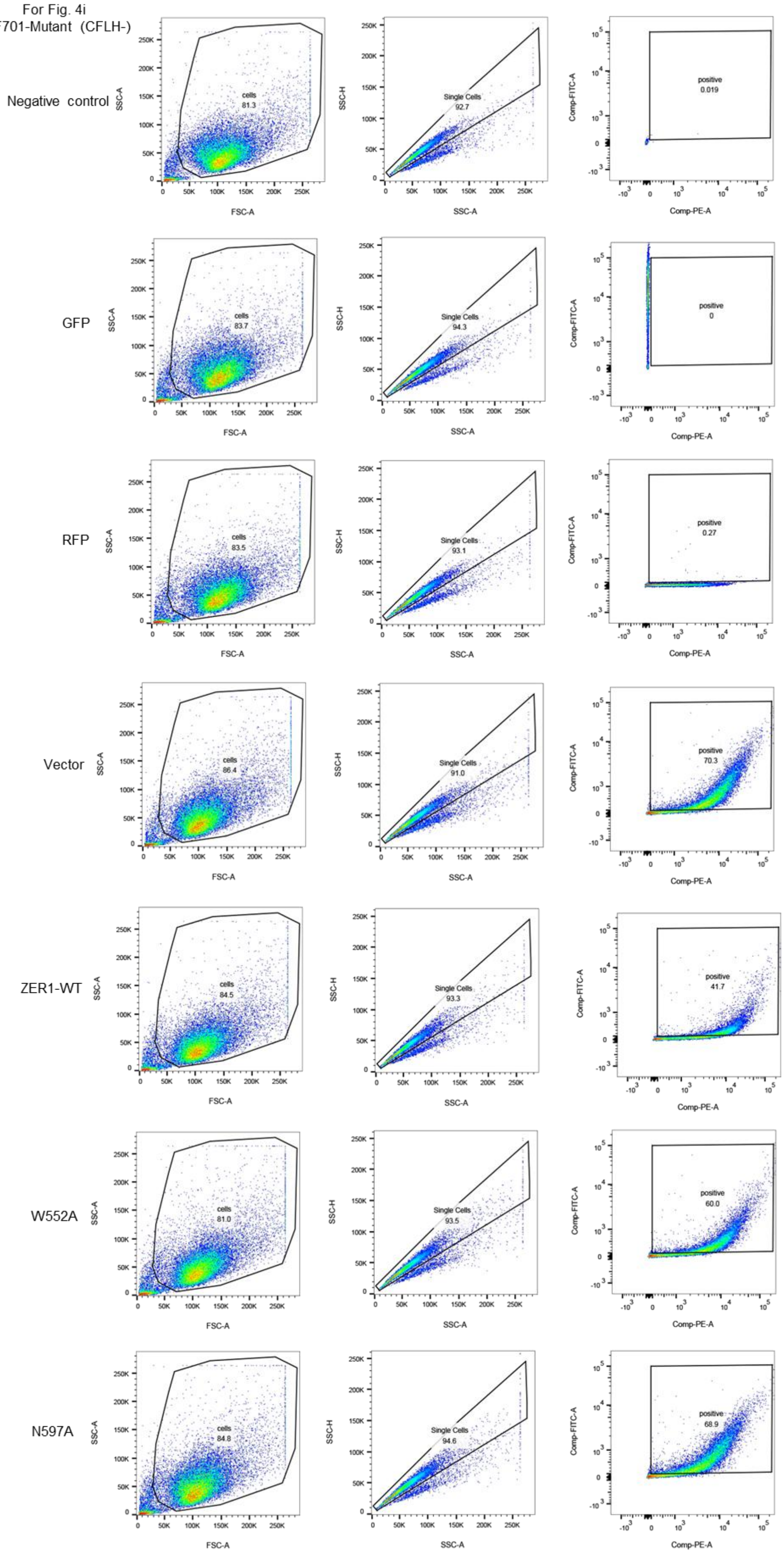

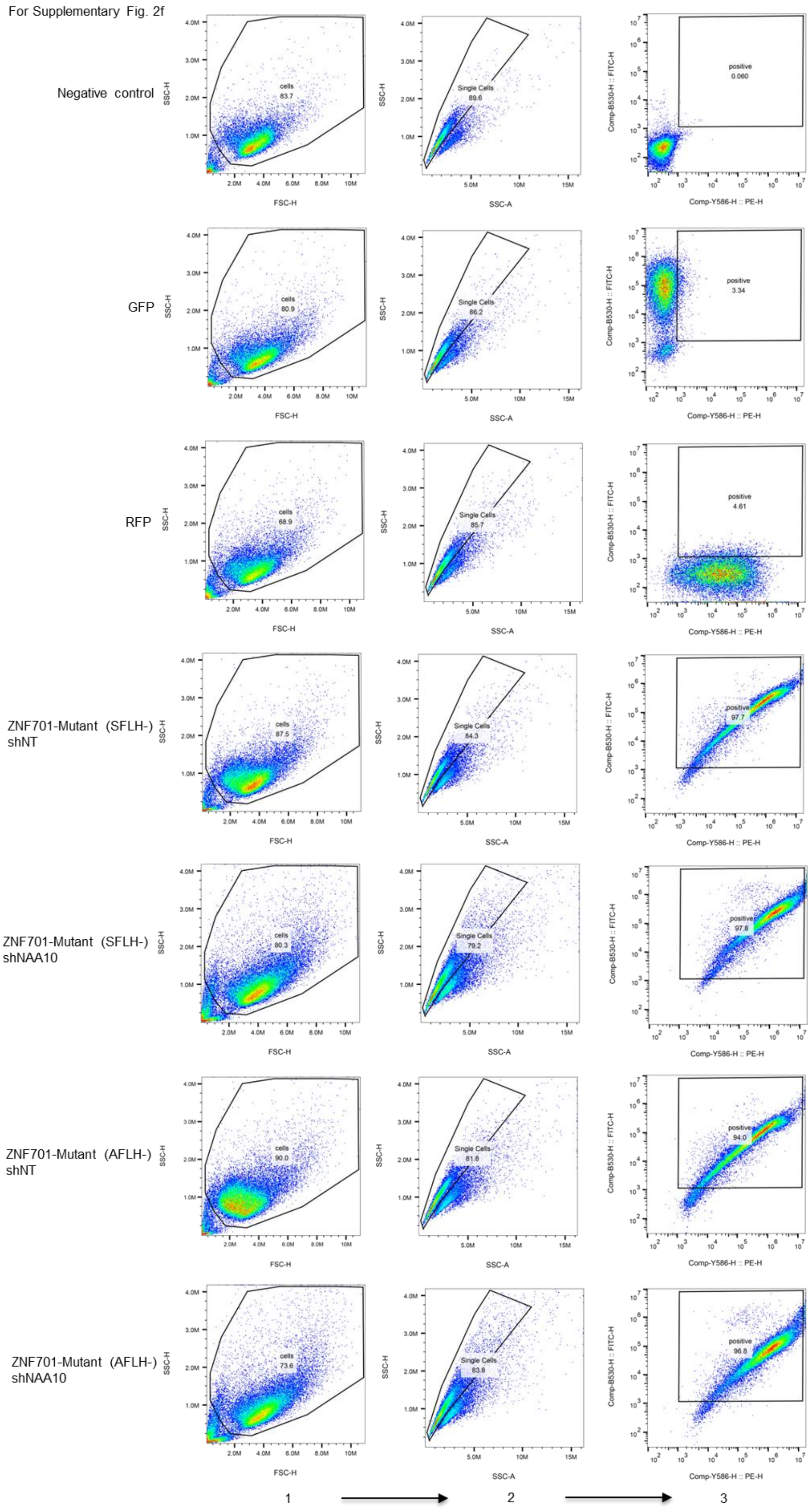

Supplementary Fig. 6 | Gating images for FACS. 1. Gate on FSC-H vs. SSC-H was set to include

all cell populations, but excluding debris; 2. Gate on SSC-A vs. SSC-H was set to exclude doublets; 3. The selected cells was calculated for GFP/DsRed ratio.
